# Eukaryotic MAGs from the NEREA observatory: expanding the coastal microbiome dataset

**DOI:** 10.1038/s41597-026-07571-y

**Published:** 2026-06-09

**Authors:** Lucia Campese, Antonio Longo, Eric Pelletier, Tom O. Delmont, Luca Ambrosino, Marco Miralto, Bruno Hay Mele, Adriana Alberti, Karine Labadie, Pedro H. Oliveira, Aude Perdereau, Patrick Wincker, NEREA coordinators, Raffaella Casotti, Fabio Conversano, Domenico D’Alelio, Francesca Margiotta, Marina Montresor, Daniele Iudicone, Daniele Iudicone

**Affiliations:** 1 Department of Integrative Marine Ecology, Stazione Zoologica Anton Dohrn, Villa Comunale, 80121 Naples, Italy; 2 Department of Biology, University of Naples Federico II, Via Cintia, 80126 Naples, Italy; 3 Génomique Métabolique, Genoscope, Institut François Jacob, Commissariat à l’Energie Atomique (CEA), CNRS, Université Evry, Université Paris-Saclay, 2 Rue Gaston Crémieux, 91057 Evry, France; 4 Research Federation for the Study of Global Ocean Systems Ecology and Evolution, FR2022/Tara GOSEE, Paris, France; 5 Department of Research Infrastructures for Marine Biological Resources, Stazione Zoologica Anton Dohrn, Villa Comunale, 80121 Naples, Italy; 6 Institute of Genetics and Biophysics “Adriano Buzzati-Traverso”, National Research Council of Italy, Via Pietro Castellino 111, 80131 Naples, Italy; 7 Università Telematica San Raffaele Roma, Via di Val Cannuta 247, 00166 Rome, Italy; 8 Genoscope, Institut François Jacob, CEA, Université Paris-Saclay, 2 Rue Gaston Crémieux, 91057 Evry, France; 9 Present Address: Université Paris-Saclay, CEA, CNRS, Institute for Integrative Biology of the Cell (I2BC), 91198 Gif-sur-Yvette, France; 10 Department of Marine Animal Conservation and Public Engagement, Stazione Zoologica Anton Dohrn, Villa Comunale, 80121 Naples, Italy

## Abstract

Marine ecosystems are hotspots of biodiversity and biogeochemical activity, yet much of their complexity remains largely inaccessible without genome-resolved data. Here we present a curated dataset of 52 eukaryotic metagenome-assembled genomes (MAGs) reconstructed from samples collected between April 2019 and January 2020 at three NEREA (Naples Ecological REsearch for Augmented observatories) sites in the Gulf of Naples. NEREA is a coastal observatory integrating physical, chemical and biological measurements with state-of-the-art metagenomics. The eukaryotic MAGs have an average completeness of ~55% and genome size of ~20 Mb. Predicted proteins were functionally annotated against UniProtKB, InterPro, and eggNOG databases, and each MAG was taxonomically classified using a curated RNA polymerase A reference dataset. The recovered MAGs encompass diverse eukaryotic lineages, primarily Ochrophyta, Chlorophyta and Haptophyta. Building on the Tara Oceans eukaryotic MAG legacy, this release represents the first reconstruction of eukaryotic MAGs from a coastal time series, enabling temporal and functional analyses of eukaryotic plankton.

## Background & Summary

**Fig. 1:**
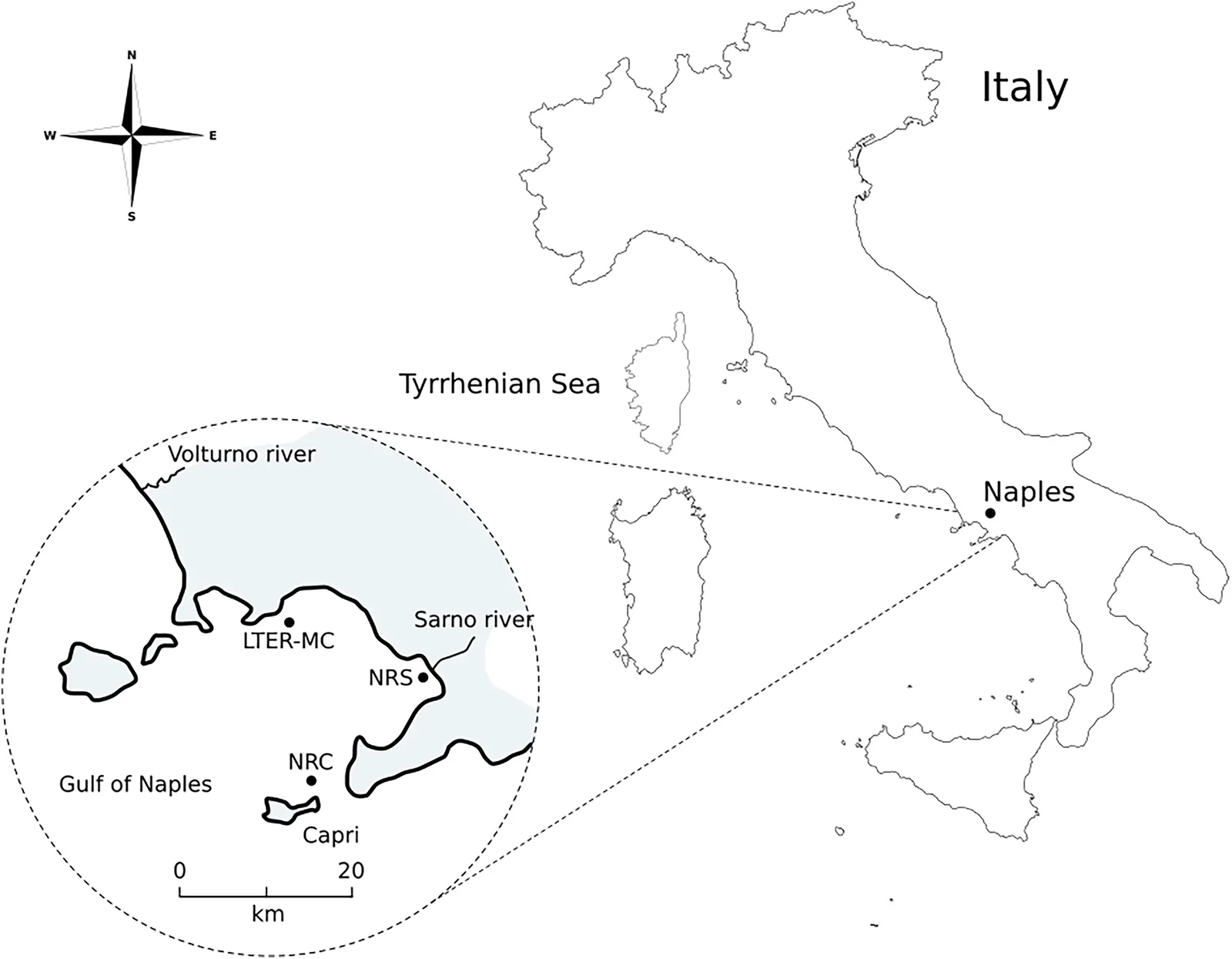
Map showing the three NEREA sampling stations: LTER-MC (Long-Term Ecological Research site MareChiara), NRS (Sarno river mouth), and NRC (Dohrn Canyon). Reused from^1^.

The NEREA (Naples Ecological REsearch for Augmented observatories) initiative captures marine microbial dynamics in the Gulf of Naples through repeated sampling using an integrative multi-omics strategy^1^. Drawing on the conceptual and methodological framework of the Tara Oceans expedition and the legacy of the Long Term Ecological Research Station-MareChiara (LTER-MC, https://deims.org/0b87459a-da3c-45af-a3e1-cb1508519411), NEREA aims to track temporal and spatial plankton dynamics, including targeted observations of key physico-chemical and biological processes. The focus is the Gulf of Naples (Fig. 1), a coastal area under strong and variable anthropogenic pressure from industrial, agricultural, transportation and eutrophication activities and also under rapid climate-change-driven alterations^2–4^.

The plankton of the Gulf of Naples and related hydrological parameters have been sampled since 1984 in the framework of the LTER-MC^5^. NEREA extends these long-term observations by integrating deep expertise in physics and oceanography, analytical chemistry (nutrients, particulate and dissolved organic matter, lipid-derived oxylipins), stable-isotope analyses (carbon and nitrogen), quantitative microscopy and flow cytometry, high-throughput metabarcoding, shotgun metagenomics, and metatranscriptomics.

MAGs enable the recovery of partial to near-complete genomes directly from environmental DNA. Unlike single-gene surveys, MAGs provide genomic context for genes belonging to the same organism, enabling a genome-wide exploration of metabolic potential.

However, reconstructing eukaryotic MAGs presents several challenges; large genome sizes, introns, repetitive elements, polyploidy, and organellar sequences complicate assembly, binning and contamination removal workflows^6^. Although pioneering efforts have recovered hundreds of marine eukaryotic MAGs, often supplemented by single-cell amplified genomes, these typically focus on high-diversity pelagic systems^7,8^. By employing co-assembly across multiple samples, automated binning coupled with rigorous manual curation, we here release 52 coastal eukaryotic MAGs, taxonomically classified across major lineages. Gene prediction was performed using the MarFERReT database^9^; functional annotation was then conducted in parallel using multiple reference databases - UniProtKB^10^, eggNOG v5^11^, and InterPro^12^ - and the results were merged to generate a non-redundant set of annotations. This collection not only enriches the global repository of marine eukaryotic MAGs, but also complements the 3,818 prokaryotic MAGs, the prokaryotic gene catalogue, the metabarcoding, environmental and biodiversity data released in^1^, therefore expanding the genomic and ecological context for integrative analyses of microbial community structure, function, and evolutionary dynamics in Mediterranean coastal systems.

## Methods

### From sampling to sequencing

Marine plankton samples were collected at three sites in the Gulf of Naples, at multiple depths and size fractions (0.22–3 µm, 3–20 µm, 20–200 µm, and 200–2000 µm), using a combination of plankton nets and Niskin bottles, with subsequent size-fractionation filtering using polycarbonate filters, following protocols adapted from the Tara Oceans expedition^13^, as detailed in^1^. In total, 59 metagenomic samples were used. Sampling depth varied among sites, spanning from surface to deep layers (up to ~800 m), depending on hydrographic conditions. DNA was co-extracted with RNA from cryogenically-ground filters using Macherey-Nagel NucleoSpin kits, quantified fluorometrically, fragmented, and subsequently prepared into metagenomic libraries. Libraries underwent end-repair, adenylation, barcode ligation, and PCR amplification before sequencing on an Illumina NovaSeq. 6000 platform (paired-end, 150 bp). Full methodological details of sampling, nucleic acid extraction, and library preparation are described in^1^. Sequencing data were processed following standard quality control procedures, including adapter removal, trimming of low-quality bases, and removal of sequencing artefacts, as described in^14^.

### From co-assembly to automatic binning

**Fig. 2:**
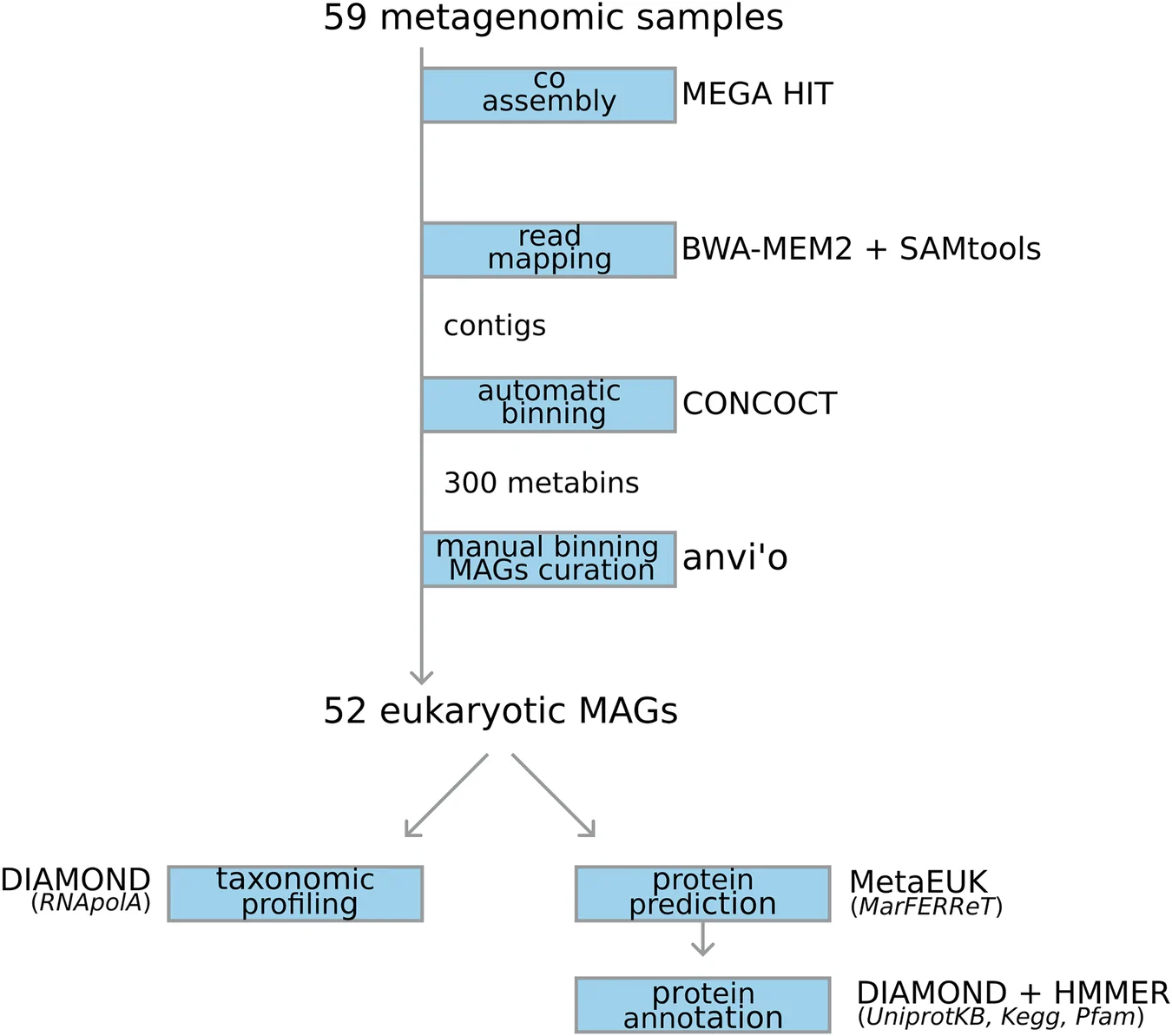
Schematic representation of the assembly, binning, and annotation pipelines used to generate the NEREA_MAGs_Euk_v1 dataset. Raw metagenomic reads from the NEREA project were co-assembled and binned into 52 eukaryotic MAGs. MAGs were then assigned to taxonomy and their proteins were predicted and functionally annotated through comparison with curated databases.

To reconstruct metagenome-assembled genomes (MAGs), we first performed a co-assembly of 59 metagenomic samples from^1^, belonging to different size classes (0.22–3 µm, n = 17; 3–20 µm, n = 15; 20–200 µm, n = 16; 200–2000 µm, n = 11). The co-assembly was performed using MEGAHIT^15^ v.2.1.9 with k-mer lengths of 41, 61, 81, 101, and 121 (Fig. 2).

Co-assembly was selected to ensure sufficient sequencing depth for the reconstruction of large eukaryotic genomes, an approach shown to be effective for the Tara Oceans data^7^. Single-sample assemblies remain informative for resolving sample-specific genomic features, and metagenomic reads can be mapped (i.e., aligned) back to contigs from these assemblies to estimate coverage across samples. However, co-assembly increases the cumulative coverage of shared genomes across samples and is therefore better suited for the recovery of eukaryotic MAGs.

The number of reads used for the co-assembly ranged from 164,100,890 to 390,363,264 per sample, reported as the sum of forward and reverse reads (R1 + R2) (total: 16,408,498,872 reads; Table S2). After assembly, the generated contigs underwent reformatting using anvi’o v.8^16^ (anvi-script-reformat-fasta) to simplify sequence headers and to remove contigs shorter than 2500 nucleotides, resulting in a refined contig file (NEREA_final.contigs.2500.fna).

Reads from individual samples were mapped back to the contigs using the BWA-MEM2 algorithm, employing a minimum percent identity threshold of 95%, as in^13^. The resulting alignments were sorted and indexed using SAMtools^17^. Mapping efficiency averaged around 20% per sample (Table S2), primarily due to the focus on larger contigs (≥2,500 nt). We then generated sample-specific contig profiles using anvi’o (anvi-profile) from the mapped BAM files, capturing coverage and single-nucleotide variant (SNV) information per contig. Each BAM file was first processed (anvi-init-bam) to meet anvi’o requirements. Individual profiles were subsequently merged (anvi-merge) into a combined dataset to leverage differential coverage information across samples.

A contig database (NEREA_final-contigs.2500.db) was created using anvi’o (anvi-gen-contigs-database), storing information such as k-mer frequencies and preliminary gene calls used for downstream binning. Genes within contigs were initially predicted using Prodigal^18^. Although read mapping and binning operate at the contig level, gene prediction is required within the anvi’o framework to identify single-copy core genes and other functional signals that are then used to estimate completeness, redundancy, and taxonomic affiliation during manual binning and curation. As anvi’o was originally developed for prokaryotic genome analysis, this step was used exclusively to support contig binning within our mixed (prokaryotic and eukaryotic) assembly. Subsequent gene prediction and functional annotation of eukaryotic MAGs were then performed using dedicated approaches tailored to eukaryotic genomes. To facilitate taxonomy assignment, we used hidden Markov models (HMMs), implemented in anvi’o (anvi-run-hmms), to identify single-copy core genes (SCGs) across the assembled contigs. These markers included standard bacterial, archaeal and eukaryotic SCGs together with additional universal markers targeting broader phylogenetic diversity, specifically the DNA-dependent RNA polymerase subunit genes^19^. This search enriched the contigs database with phylogenetically informative marker gene occurrences.

To delineate potential genomes, we employed automatic binning via the CONCOCT^20^ algorithm (anvi-cluster-contigs), which clusters contigs based on sequence composition and differential coverage across samples (Fig. 2). Following the Tara Oceans eukaryotic MAGs approach^7^, we constrained CONCOCT to generate 300 clusters, a number intentionally set well below the expected total number of genomes in the assembly (across all domains of life) in order to minimize fragmentation of individual genomes. This under-clustering strategy results in broad initial clusters (“metabins”), which may include contigs from multiple organisms. These metabins were subsequently subjected to manual binning and curation within the anvi’o interactive interface to resolve individual eukaryotic MAGs.

### Manual binning, MAG curation and taxonomic annotation

**Fig. 3:**
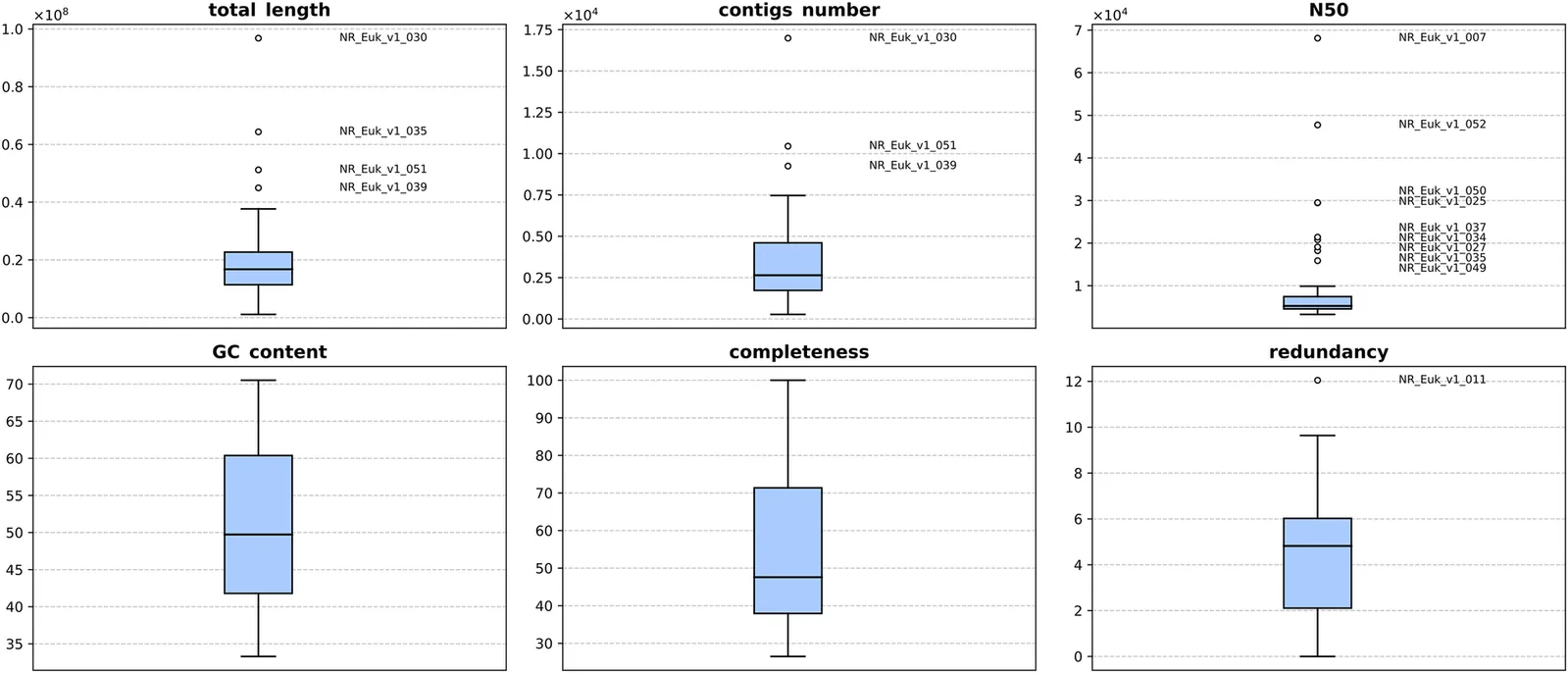
Distribution of assembly and quality metrics across NEREA eukaryotic MAGs. Each boxplot represents the variability of total length (bp), contig number, N50 (bp), GC content, completeness (%), and redundancy (%), highlighting overall assembly consistency and outlier genomes.

We used the RNA polymerase subunit A (RNApolA) marker genes previously identified on the assembled contigs to assess the taxonomic affiliation of the 300 metabins. RNApolA sequences retrieved from these metabins were aligned with DIAMOND v.2^21^ against a curated reference database of taxonomically annotated RNApolA sequences^22^. Based on the taxonomic affiliation of these marker genes, 104 metabins showed a clear eukaryotic signal and were subsequently inspected in anvi’o for the manual extraction and curation of individual eukaryotic MAGs. Completeness and redundancy values were estimated in anvi’o using a set of eukaryotic single-copy core genes (SCGs) derived from BUSCO^16^. Specifically, completeness was calculated based on the BUSCO_83_Protista marker set available in anvi’o, as the percentage of the 83 protist SCGs detected at least once within a MAG. Redundancy was reported from the same SCGs set as the percentage of markers detected in more than one copy within a MAG, and was used as a proxy for potential contamination or mixed bins. Only MAGs with a minimum completeness of 25% were retained. Redundancy was controlled to maintain an acceptable balance with completeness, with a maximum tolerated value of 12%. As a result, a total of 52 eukaryotic MAGs were recovered from the selected eukaryotic metabins (Fig. 2) and their quality features are reported in Fig. 3.

Taxonomic annotation of the final set of eukaryotic MAGs was refined by aligning their RNA polymerase subunit A (RNApolA) sequences against the curated RNApolA reference database using DIAMOND. In contrast to the initial annotation step, which was based on RNApolA markers retrieved from metabins that could still contain multiple genomes, the second round of annotation was performed on manually curated MAGs and therefore yielded a more robust taxonomic assessment. Taxonomic assignment was based on the best DIAMOND hit to the curated RNApolA reference database, using the following percent identity thresholds: ≥ 80% for genus, 65–80% for family, 50–65% for order, and < 50% for class. Even when identity exceeded 95%, assignments were conservatively limited to the genus level. When the identity threshold supported a given rank but the best-matching reference sequence was annotated only at a higher taxonomic level, we conservatively retained the highest rank available in the reference database. One MAG (NR_Euk_v1_017) showed only 34% identity to the closest reference sequence and was therefore classified as unknown, although its eukaryotic origin was supported by the marker-gene evidence (Table S1).

The dataset includes representatives of major phyla such as Ochrophyta, Chlorophyta, Arthropoda, Haptophyta, Chordata, MAST, Cercozoa, Cryptophyta and Dinoflagellata, encompassing the most dominant microbial eukaryotic groups in marine ecosystems (Table 1). Read mapping across size-fractionated metagenomic samples further showed that different taxonomic classes were preferentially represented in different size fractions, as reflected by the fraction in which MAGs from each class reached their highest average coverage (Table 1; Table S2). These patterns were broadly consistent with the expected size distribution of the corresponding taxa, with several diatom and metazoan classes showing highest coverage in the 20–200 µm fraction, while smaller pico- and nanoeukaryotic groups were more strongly represented in the 0.22–3 µm or 3–20 µm. A small number of MAGs were additionally assigned to metazoan lineages (e.g., zooplankton taxa), reflecting the inclusion of planktonic life stages captured during sampling and are retained to provide a comprehensive representation of the planktonic community.

**Table 1:** Taxonomic distribution and representative size fractions in the NEREA eukaryotic MAG collection.

Phylum	Class	Number of MAGs	Representative size fraction
Ochrophyta	Mediophyceae	13	20–200 µm
Ochrophyta	Chrysophyceae	6	0.22–3 µm
Ochrophyta	Bacillariophyceae	3	20 –200 µm
Ochrophyta	Pelagophyceae	3	3–20 µm
Ochrophyta	Dictyochophyceae	2	3–20 µm
Ochrophyta	Bolidophyceae	1	0.22–3 µm
Chlorophyta	Mamiellophyceae	7	3–20 µm
Chlorophyta	Chloropicophyceae	2	20–200 µm
Haptophyta	Prymnesiophyceae	3	3–20 µm
Arthropoda	Hexanauplia	3	20–200 µm
Chordata	Appendicularia	3	20–200 µm
MAST	MAST-7	2	3–20 µm
MAST	MAST-4-Clade-A	1	3–20 µm
Cercozoa	Chlorarachniophyceae	1	0.22–3 µm
Cryptophyta	Cryptophyceae	1	3–20 µm
Dinoflagellata	Dinophyceae	1	3–20 µm

The representative size fraction indicates the size fraction in which MAGs from each class reached their highest average coverage.

### Protein prediction and functional annotation

To determine the most suitable reference database for gene prediction, we applied the easy-predict module of MetaEuk v.7^23^ to the four NEREA MAGs with the highest completeness based on the BUSCO_83_Protista marker set (NR_Euk_v1_025: *Micromonas*, NR_Euk_v1_034: *Chaetoceros*, NR_Euk_v1_050: *Pseudo-nitzschia*, NR_Euk_v1_052: *Ostreococus*). Protein predictions were conducted using multiple reference databases, including UniProtKB and SwissProt v.2025_03^10^, NCBI NR v.2025_03^24^, EukProt v.3^25^, and MarFERReT^9^. Among these, MarFERReT consistently produced the highest number of predicted proteins. Based on this comparative analysis and its relevance as a marine-focused resource, MarFERReT was selected as the optimal reference database for final gene prediction across all NEREA MAGs.

**Fig. 4:**
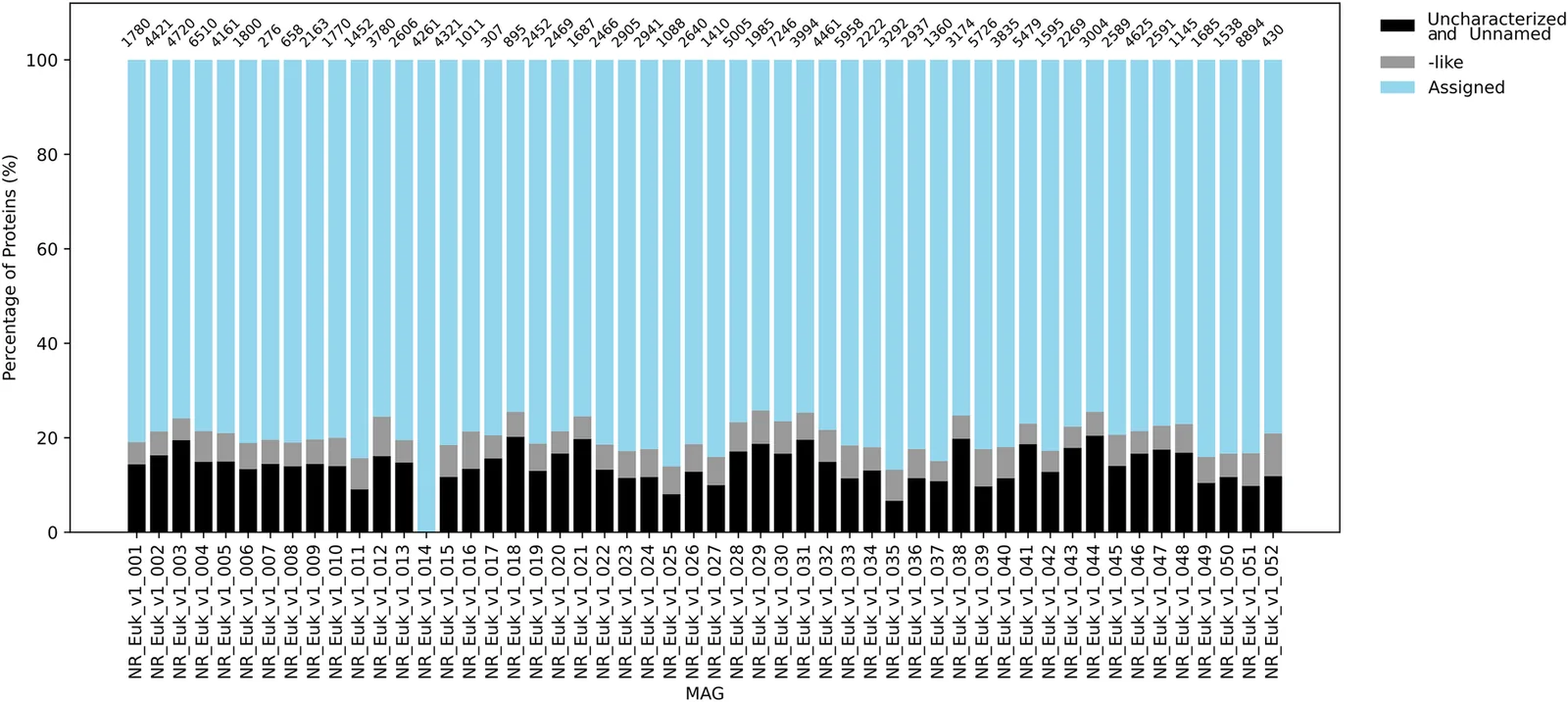
Stacked bar plots showing the relative composition of protein annotations across the 52 eukaryotic MAGs based on best e-value hits. Each bar represents one MAG, displaying the proportion of “Assigned” (light blue), “–like” (gray), and “Uncharacterized and Unnamed” (black) proteins. Percentages are calculated over the total number of unique genes retained after filtering.

Functional annotation of predicted protein sequences was performed using three complementary approaches: (i) direct sequence similarity searches against the UniProtKB database were conducted using DIAMOND, providing protein-level functional descriptions based on the best hit for each query; (ii) orthology- and functional categorised annotations were assigned using eggNOG-mapper v.2^26^ with the eggNOG v.5.0.2^11^ database, yielding orthologous groups, COG functional categories, Gene Ontology (GO) terms, and KEGG Orthology (KO) identifiers; (iii) domain- and family-level functional annotation was performed using InterProScan v.5.74–105^27^, which identified conserved protein domains, families, and functional sites based on the integrated InterPro collection of protein signature databases, including Pfam and InterProID. The outputs from these approaches were merged based on the protein identifiers to generate a final comprehensive annotation table in TSV format for each MAG, providing a complete list of functional assignments. Proteins with multiple annotations were consolidated by retaining only the best hit (lowest e-value) per gene. The resulting dataset was classified into three major categories based on annotation content: “Uncharacterized and Unnamed” proteins lacking informative functional or family description; “- like” proteins with weak or tentative homology to known sequences; “assigned” proteins with clearly defined and confidently attributed functions (Fig. 4).

## Data Records

Nucleotidic FASTA files for the 52 MAGs generated in this study are available in the European Nucleotide Archive (ENA) under BioProject^28^ (https://www.ebi.ac.uk/ena/browser/view/PRJEB98715), within the umbrella project NEREA Augmented Observatory. The co-assembly FASTA file from which the MAGs were extracted is available in Zenodo^22^ (https://zenodo.org/records/20327956), together with a text file describing the analysis pipeline, the script used for read mapping, the RNApolA FASTA sequences with their associated metadata, and the supplementary tables. Predicted protein sequences for the 52 eukaryotic MAGs have been deposited in Zenodo^29^ (https://zenodo.org/records/1737624) as amino acidic FASTA files, while the merged functional annotations of the corresponding predicted proteomes are available in a separate Zenodo record^30^ (https://zenodo.org/records/17403867) as 52 individual tables.

## Technical Validation

Quality validation of the genomic resources released in this Data Descriptor focused on the computational workflow used to generate the NEREA co-assembly and eukaryotic MAGs. High-resolution coverage profiles were computed across all metagenomes included in the co-assembly and used in combination with automatic binning. Each resulting bin was then manually inspected and refined to enable the resolution of composite bins and the removal of potential contaminants. Taxonomic assignments were validated by cross-referencing phylogenetic marker genes and DIAMOND-based similarity searches against curated marine reference databases. For gene prediction, we benchmarked MetaEuk results obtained using multiple reference databases (UniProtKB, SwissProt, NCBI NR, EukProt, and MarFERReT) on the four most complete NEREA MAGs. Taken together, these procedures ensure that the co-assembly and MAGs released here constitute high-quality, well-validated genomic resources suitable for downstream ecological and evolutionary analyses.

## Usage Notes

The eukaryotic MAGs and co-assembly released here can be directly integrated into comparative genomics, functional profiling, and metatranscriptomic read recruitment analyses. Their compatibility with the broader NEREA multi-omics framework, which includes environmental parameters, microscopy, flow cytometry, metabarcoding, and prokaryotic MAG datasets published in^1^, enables cross-layer investigations of plankton diversity and ecosystem dynamics. As with any metagenome-derived resource, MAGs may underrepresent low-abundance or highly fragmented genomes, and co-assembly strategies can smooth strain-level diversity. These aspects should be considered when interpreting fine-scale ecological patterns.The NEREA observatory is ongoing, and future data releases will expand the genomic catalogue and incorporate metatranscriptomic datasets, facilitating the study of gene expression dynamics and seasonal processes at higher resolution.

## Supplementary information

Supplementary Information

Supplementary Information

## Data Availability

All data described in this study are publicly available. Reconstructed MAGs have been deposited in the European Nucleotide Archive (ENA) PRJEB98715. Additional derived datasets are available through Zenodo, including the co-assembly summary and MAG taxonomic overview (https://doi.org/10.5281/ZENODO.20327956), predicted protein sequences (https://doi.org/10.5281/ZENODO.17376243), and functional annotations of the predicted proteomes (https://doi.org/10.5281/ZENODO.17403867).

## Code availability

The code and command-line workflow used in this study are available at^22^. The repository includes pipeline_nerea_mags.txt, a text file documenting the commands and analysis steps used to generate the MAG catalog, together with the map.sh script used for read mapping.
